# Dynamic multicolor protein labeling in living cells†
†Electronic supplementary information (ESI) available. See DOI: 10.1039/c7sc01364g



**DOI:** 10.1039/c7sc01364g

**Published:** 2017-05-30

**Authors:** Chenge Li, Marie-Aude Plamont, Hanna L. Sladitschek, Vanessa Rodrigues, Isabelle Aujard, Pierre Neveu, Thomas Le Saux, Ludovic Jullien, Arnaud Gautier

**Affiliations:** a École Normale Supérieure , PSL Research University , UPMC Univ Paris 06 , CNRS , Département de Chimie , PASTEUR , 24 rue Lhomond , 75005 Paris , France; b Sorbonne Universités , UPMC Univ Paris 06 , ENS , CNRS , PASTEUR , 75005 Paris , France . Email: ludovic.jullien@ens.fr ; Email: arnaud.gautier@ens.fr; c Cell Biology and Biophysics Unit , European Molecular Biology Laboratory , Meyerhofstr. 1 , D-69117 Heidelberg , Germany

## Abstract

Yellow Fluorescence-Activating and absorption-Shifting Tag (Y-FAST, hereafter called FAST) is a 14 kDa protein tag giving a bright green-yellow fluorescent complex upon interaction with the fluorogenic dye 4-hydroxy-3-methylbenzylidene rhodanine (HMBR). Here, we report a collection of fluorogens enabling tuning of the fluorescence color of FAST from green-yellow to orange and red. Beyond allowing the multicolor imaging of FAST-tagged proteins in live cells, these fluorogens enable dynamic color switching because of FAST’s reversible labeling. This unprecedented behavior allows for selective detection of FAST-tagged proteins in cells expressing both green and red fluorescent species through two-color cross-correlation, opening up exciting prospects to overcome spectral crowding and push the frontiers of multiplexed imaging.

## Abstract 2

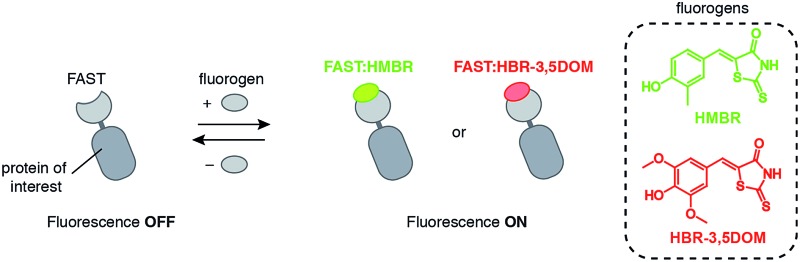
Engineered fluorogenic dyes enable the dynamic switching of the fluorescence color of Fluorescence-Activating and absorption-Shifting Tag (FAST).

## Introduction

Imaging the dynamics of proteins in living cells is essential for deciphering biological processes. A common strategy for imaging proteins is to fuse them to peptide or protein sequences that provide fluorescence, such as autofluorescent proteins^1,2^ or self-labeling tags^3–5^ (such as a SNAP-tag^6,7^/CLIP-tag^8^ and Halo-tag^9^) that can be labeled specifically with chemical probes. A related strategy is to use protein tags that can generate fluorescence upon the interaction and activation of fluorogenic compounds (so-called fluorogens).^10,11^ Fluorogen-activating proteins (FAPs) that are able to specifically bind to fluorogens, such as malachite green and thiazole orange, and activate their fluorescence have been developed from single-chain antibodies.^12,13^ Because fluorogens exhibit very low fluorescence background in cells, proteins can be imaged with high contrast without the need for washing the fluorogen excess.

The concept of fluorogenic reporters was recently pushed one step further with the design of a tunable genetically encodable reporter called Yellow Fluorescence-Activating and absorption-Shifting Tag (Y-FAST, hereafter called FAST) that not only can be switched on by the addition of a fluorogenic ligand, but can also be switched off rapidly by the removal of the fluorogen through washing.^14^ This fluorescence on/off switch behavior was made possible by designing a system displaying dynamic reversible fluorogen binding, characterized in particular by a high off-rate constant enabling rapid complex dissociation upon fluorogen removal. FAST is a 14 kDa variant of the photoactive yellow protein (PYP) engineered to specifically bind and activate the fluorescence of the fluorogenic dye HMBR. HMBR undergoes two spectroscopic changes upon binding, ensuring high labelling selectivity. While unbound HMBR mainly deexcites from the excited state through non-radiative processes, bound fluorogen undergoes a very large increase of the fluorescence quantum yield because of immobilization within the FAST cavity. Moreover, FAST binds HMBR in its anionic form, minor form in solution at pH 7.4, leading to an apparent 80 nm absorption red shift. FAST is thus an acronym that echoes back to its fast exchange dynamics and stands for Fluorescence-Activating and absorption-Shifting Tag, highlighting the two spectroscopic changes ensuring labeling selectivity.

**Fig. 1:**
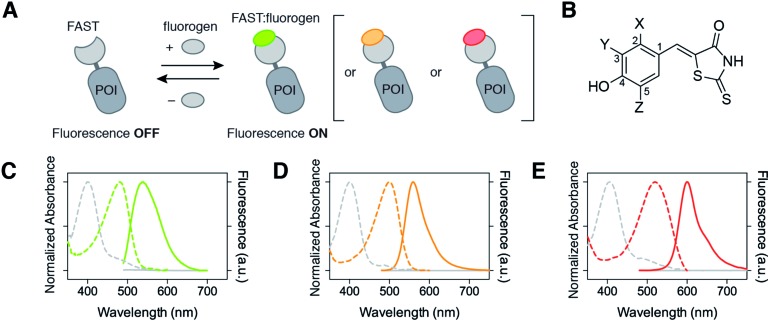
Multicolor imaging of FAST-tagged proteins. (A) The fluorescence color of FAST can be adapted by changing the fluorogen (POI: protein of interest). (B) Structures of the fluorogens discussed in this study: HMBR (X = H, Y = Me, Z = H), HBR-3E (X = H, Y = Et, Z = H), HBR-2,5DM (X = Me, Y = H, Z = Me), HBR-3,5DM (X = H, Y = Me, Z = Me), HBR-3OM (X = H, Y = OMe, Z = H), HBR-3OE (X = H, Y = OEt, Z = H), and HBR-3,5DOM (X = H, Y = OMe, Z = OMe). (C–E) The absorption (dashed line, left axis) and emission (solid line, right axis) spectra of the free fluorogens (grey line) and FAST:fluorogen complexes (colored lines) in pH 7.4 phosphate buffer saline at 25 °C ((C) HMBR, (D) HBR-3,5DM, and (E) HBR-3,5DOM). Note that the shoulders at 480 nm on the absorption spectra of the free fluorogens correspond to anionic fluorogens present in low amounts at pH 7.4 (see also Fig. S1 and Table S1†).

The complex FAST:HMBR fluoresces green-yellow light (*λ*_em_ = 540 nm) upon blue light excitation. In order to provide investigators with the possibility to tune the spectral properties of FAST with their imaging conditions, we sought alternative fluorogens able to bind FAST and form complexes with novel fluorescence properties. We explored if changing the structure of HMBR could shift both the excitation and emission of the tag:fluorogen complex to the red edge of the visible spectrum (Fig. 1A). We report a collection of fluorogen analogs with various spectral properties. We present in particular HBR-3,5DOM (4-hydroxy-3,5-dimethoxybenzylidene rhodanine), a new fluorogen that forms a tight complex with FAST that fluoresces red light under green light excitation. This allows for the choice of the FAST color by choosing the appropriate fluorogen, providing an experimental versatility that is not encountered with autofluorescent proteins. We also show that we could exploit dynamic fluorogen binding to switch the color from red to green or green to red by simply changing the fluorogen used. The ability to dynamically switch color allowed us to further develop a new strategy to selectively image FAST-tagged proteins in a spectrally crowded environment. The spectral overlap between fluorophores limits most experiments to the simultaneous observation of three or four targets, thus preventing highly multiplexed imaging. Here, we show that one can overcome spectral crowding using kinetic filtering based on dynamic color switching and two-color cross-correlation.

## Results and discussion

The HMBR fluorogen is composed of an electron-donating phenol ring conjugated to an electron-withdrawing rhodanine heterocycle. This push–pull structure deexcites non-radiatively in solution but relaxes radiatively to the ground state within the FAST cavity. The substituents on the aromatic ring are thus *a priori* key determinants of the fluorescence efficiency, as they can play a major role in the (im)mobility of the fluorogen within the FAST cavity. In addition, the spectral properties of the push–pull systems, such as HMBR and its analogs, are directly related to the electron donating or withdrawing ability of the donor and acceptor groups, as they affect the energy of the molecular orbitals. Changing the electron-donating properties of the phenol moiety through adequate substitution can thus affect the spectroscopic properties. In particular, here we can anticipate that the stronger the electron donating ability of the donor part, the more red-shifted the absorption and emission. We thus examined the role of the substituents on the aromatic ring. Fig. 1B gives the structure of the different analogs obtained by condensation of the rhodanine heterocycle with appropriate aromatic aldehydes. The described fluorogens bound tightly to FAST with dissociation constants below 1 μM, enabling to anticipate efficient and selective FAST labeling (Table 1). In addition, all the fluorogenic analogs underwent a very large fluorescence increase (from 200 up to 600 fold) and absorption red-shift (between 80 and 110 nm) upon FAST binding (Table 1). This latter absorption red-shift is due to the binding-induced deprotonation of the fluorogens, which are all mainly protonated in solution at physiological pH (Table S1 and Fig. S1†).

**Table 1:** Physicochemical properties of FAST:fluorogen complexes in PBS pH 7.4. The abbreviations are as follows: *λ*_abs_, the wavelength of maximal absorption; *λ*_em_, the wavelength of maximal emission; *ε*, the molar absorption coefficient at *λ*_abs_; Δ*λ*_abs_ = *λ*_abs,bound_ – *λ*_abs,unbound_, the absorption red-shift upon FAST binding; *φ*, the fluorescence quantum yield; *F*_bound_/*F*_unbound_, the fluorescence activation upon FAST binding; brightness = *ε* × *φ*; *K*_D_, the thermodynamic dissociation constant. The spectroscopic data of mCherry^15^ are given for comparison

Complex	*λ*_abs_ (nm)	Δ*λ*_abs_ (nm)	*λ*_em_ (nm)	*ε* (mM^–1^ cm^–1^)	*φ* (%)	*F*_bound_/*F*_unbound_	Brightness	*K*_D_ (μM)
FAST:HMBR	481	80	540	45	23	300	10 300	0.13
FAST:HBR-3E	481	81	543	50	13	300	6500	0.08
FAST:HBR-2,5DM	494	88	552	50	29	430	14 500	0.008
FAST:HBR-3,5DM	499	98	562	48	49	660	23 500	0.08
FAST:HBR-3OM	494	91	561	40	36	350	14 400	0.31
FAST:HBR-3OE	497	94	562	41	17	230	7000	0.27
FAST:HBR-3,5DOM	518	112	600	39	31	220	12 000	0.97
mCherry	587		610	72	22		15 800	

We observed various spectroscopic behaviors. HBR-2,5DM, HBR-3,5DM, HBR-3OM and HBR-3OE formed complexes with ∼15–20 nm red-shifted absorption (*λ*_abs_ from 494 to 499 nm) and ∼10–20 nm red-shifted emission (*λ*_em_ from 552 to 562 nm) relative to FAST:HMBR (see also Fig. 1C and D). Of particular interest, FAST:HBR-3,5DM exhibits a fluorescence quantum yield of 49%, and an overall brightness 2.5-fold higher than the original FAST:HMBR complex. The greatest spectral shift was observed with HBR-3,5DOM, which bears two electron-donating methoxy substituents in the *ortho* position of the hydroxyl group. The resulting complex, FAST:HBR-3,5DOM, displayed a 40 nm red-shifted absorption (*λ*_abs_ = 518 nm) and a 60 nm red-shifted emission (*λ*_em_ = 600 nm) (see also Fig. 1E). These spectral modifications shift the absorption to the green and the emission to the red, thus providing a new color for the fluorescence imaging. Remarkably, FAST:HBR-3,5DOM is as bright as the red fluorescent protein mCherry.

**Fig. 2:**
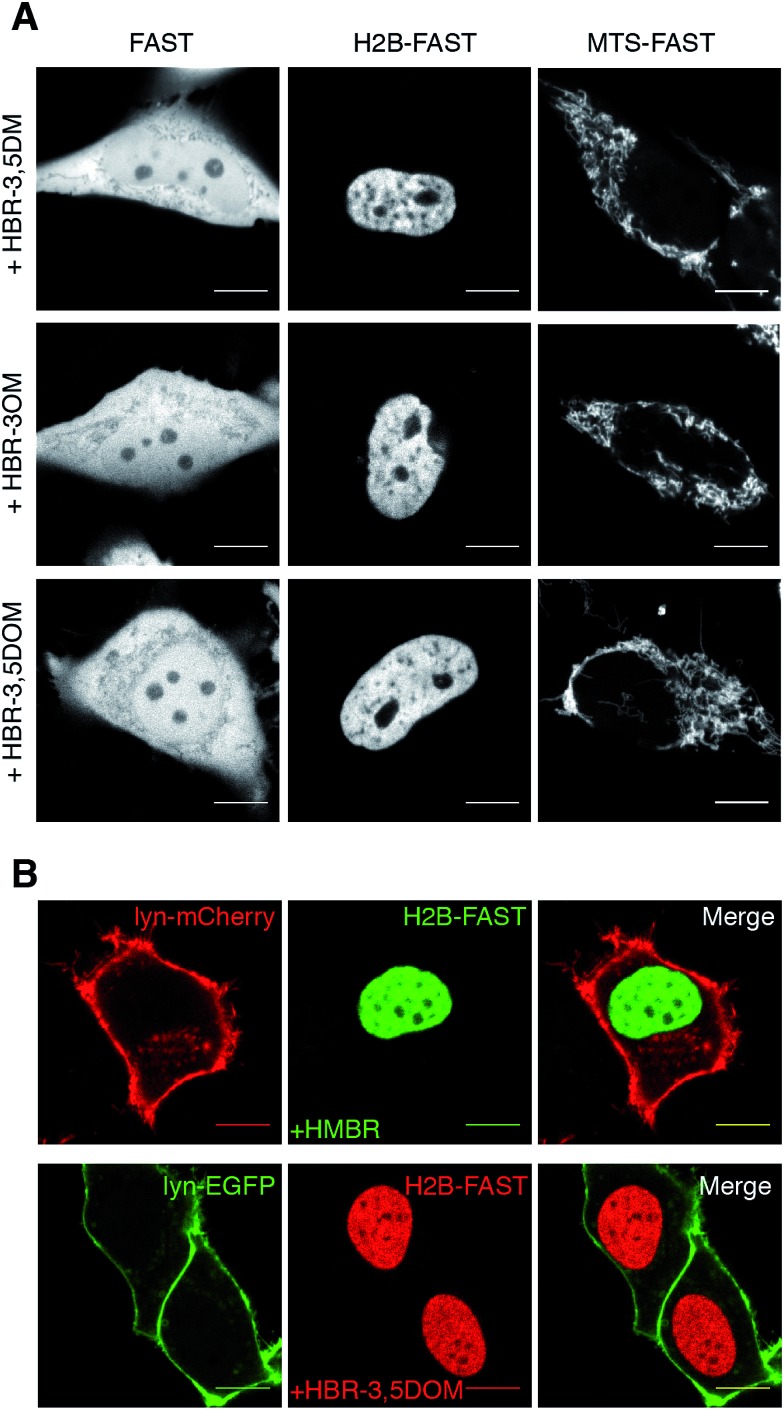
Multicolor labeling of the FAST-tagged proteins in live cells. (A) Confocal micrographs of HeLa cells expressing cytoplasmic FAST, nuclear H2B-FAST and mitochondrial MTS-FAST and labeled with 5 μM HBR-3,5DM, HBR-3OM and HBR-3,5DOM (HBR-3OM, HBR-3,5DM: Ex/Em 488/493–797 nm; HBR-3,5DOM: Ex/Em 514/519–797 nm). (B) Confocal micrographs of live HeLa cells co-expressing lyn-mCherry/H2B-FAST (top) and lyn-EGFP/H2B-FAST (bottom) labeled with 5 μM HMBR or HBR-3,5DOM, respectively (green channel Ex/Em 488/493–575 nm; red channel Ex/Em 543/578–797 nm). The scale bars are 10 μm.

We next examined if our new fluorogenic analogs could efficiently label FAST-tagged proteins in living cells. We first verified that they were non-toxic for mammalian cells (Fig. S2†). Then, we expressed in HeLa cells (i) FAST, (ii) FAST fused to histone H2B for nuclear localization, and (iii) FAST fused to the mitochondrial targeting sequence (MTS) from subunit VIII of human cytochrome c oxidase. The cells were treated with media containing 5 μM of fluorogen and imaged by confocal microscopy (Fig. 2A). We visualized the various FAST fusions in their expected localization, in agreement with the specific fluorogenic labeling.

Given that the fluorescence color of FAST can be either green-yellow (with HMBR) or red (with HBR-3,5DOM), we next showed that FAST could be imaged in the presence of either the red fluorescent protein mCherry or the green fluorescent protein EGFP by adding the complementary fluorogen. We expressed FAST fused to H2B for nuclear targeting together with either mCherry or EGFP fused to lyn N-terminal domain (lyn) for membrane anchoring. The cells co-expressing FAST and mCherry fusions were treated with media containing 5 μM HMBR, while the cells co-expressing FAST and EGFP fusions were treated with media containing 5 μM of HBR-3,5DOM. By using a dual 488/543 nm excitation and variable spectral detection, FAST:HMBR could be separated spectrally from mCherry, while FAST:HBR-3,5DOM could be distinguished from EGFP (Fig. 2B), demonstrating the versatility of FAST and the possibility to easily adapt its color to the spectral conditions of the experiment. An additional interesting feature of FAST:HBR-3,5DOM is its high Stokes shift (82 nm), which allowed us to image the FAST-tagged proteins together with the EGFP fusions using a single 488 nm excitation source and two separate spectral regions (Fig. S3†), opening up interesting prospects for live cell imaging as single excitation simplifies the imaging protocols, results in a gain of temporal resolution and reduces the phototoxicity.

**Fig. 3:**
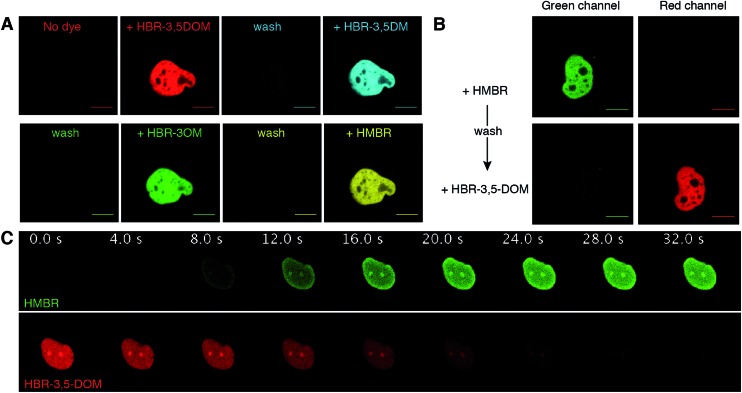
Reversible FAST labeling. (A) Confocal micrographs of a HeLa cell expressing nuclear H2B-FAST sequentially labeled with HBR-3,5DOM (red), HBR-3,5DM (cyan), HBR-3OM (green) and HMBR (yellow) (HBR-3OM, HBR-3,5DM, HMBR: Ex/Em 488/493–797 nm; HBR-3,5DOM: Ex/Em 514/519–797 nm). The labeling was reversed by removing the fluorogen-containing solutions followed by a wash with pH 7.4 PBS. (B) Confocal micrographs of a HeLa cell expressing nuclear H2B-FAST sequentially labeled with HMBR and HBR-3,5DOM (green channel: Ex/Em 488/493–550 nm; red channel: Ex/Em 543/600–797 nm). The color switch was performed by first washing away the excess of HMBR with pH 7.4 PBS, and then adding HBR-3,5DOM solution. (C) Time series of a live HeLa cell expressing H2B-FAST labeled with HBR-3,5DOM upon the replacement of the medium with a HMBR solution (single excitation at 488 nm; HMBR emission channel 493–538 nm; HBR-3,5-DOM emission channel 649–797 nm). HMBR was added at *t* = 0 s. See also Movie S1.† (A–C) The concentration of the fluorogen solutions was 5 μM. The cells were grown in a minifluidic channel enabling easy solution replacement. The scale bars are 10 μm.

A unique feature that distinguishes FAST from other labeling techniques is the possibility to rapidly reverse the labeling by washing the fluorogen away. We thus verified that we kept this property when using the new fluorogenic analogs. We expressed FAST fused to histone H2B in mammalian cells. Iterative labeling/washing with the new fluorogens allowed us to demonstrate that FAST conserved its ability to be switched on and off at will by fluorogen addition and removal (Fig. 3A). Moreover, as HMBR and HBR-3,5DOM give complexes of different fluorescence colors, FAST could be switched from one color to the other by washing away one fluorogen and adding the other one (Fig. 3B). The color switch could be done furthermore in a dynamic fashion in a few seconds simply by changing the medium content (Fig. 3C and Movie S1†).

**Fig. 4:**
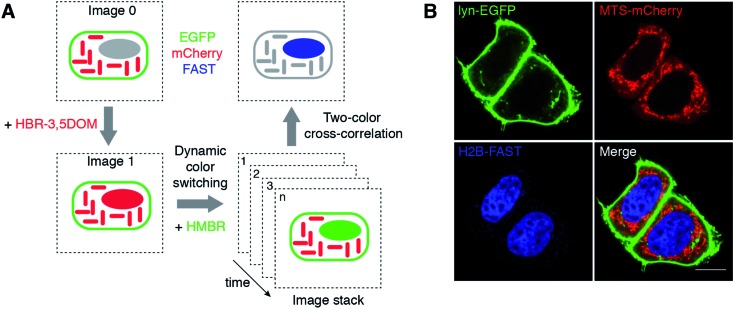
Selective imaging. (A) Measuring the degree of anti-correlation of the green and red fluorescence signals upon dynamic color switching by two-color cross-correlation is an effective method to quantitatively extract the signal of FAST in a spectrally crowded environment containing green and red fluorescent species. (B) The micrographs show live HeLa cells co-expressing three proteins: lyn-EGFP, MTS-mCherry and H2B-FAST. The lyn-EGFP (green) and MTS-mCherry (red) images are confocal micrographs obtained in the absence of fluorogen, while the H2B-FAST (blue) image results from two-color cross-correlation upon the HBR-3,5DOM-to-HMBR exchange (green channel Ex/Em 488/493–575 nm; red channel Ex/Em 543/578–797 nm). Fig. S4 and Movie S2† show the HBR-3,5DOM-to-HMBR exchange. The scale bars are 10 μm.

The ability to dynamically switch color by fluorogen exchange provides FAST with a unique signature that can be exploited to push multiplexed imaging. Modern biology needs to be able to observe multiple targets (>10–50) to study biological processes in all their complexity.^16^ Despite the large collection of probes and biosensors available, distinguishing a large number of labels remains one of the biggest challenges in imaging. A major obstacle to multiple observations is the spectral overlap between fluorophores, which limits most experiments to the observation of three or four targets. New approaches for highly multiplexed imaging relying on parameters other than spectral discrimination are thus essential if one wants to be able to better analyze multiparametric cellular processes. We thus explored if the unique signature provided by dynamic color switching could provide a solution for overcoming spectral crowding. Upon dynamic color switching, HMBR and HBR-3,5DOM are fluorescent in a temporally anti-correlated manner, as they both bind to the same tag. Kinetic information has been previously exploited to image selectively reactive fluorescent species in the presence of a high fluorescence background.^17–19^ We thus anticipated that measuring the degree of anti-correlation of the green and red fluorescence signals should enable the extraction of FAST contribution in the presence of the green and red fluorescent species – even though spectral discrimination is impossible in these conditions – exploiting the fact that only the green and red signals originating from FAST would interconvert (Fig. 4A). To evaluate this idea, we used time-lapse confocal microscopy to image HeLa cells co-expressing FAST (fused to histone H2B), mCherry (fused to a mitochondrial targeting sequence (MTS)) and EGFP (fused to lyn for membrane anchoring) upon the dynamic fluorogen exchange (Fig. 4B, S4A and B and Movie S2†). The cross-correlation of the temporal evolution of the green and red fluorescence signals allowed us to selectively extract the contribution of H2B-FAST and fully eliminate the signals of mitochondrial mCherry and membrane-targeted EGFP (Fig. 4B). Note that we chose different localizations for FAST, mCherry and EGFP to assign the proteins without any ambiguity in this proof-of-principle experiment, but the approach is generic and can of course be expanded to co-localized proteins. This experiment demonstrates that dynamic color switching and two-color cross-correlation allow unveiling of the presence of FAST in a spectrally crowded environment containing both green and red fluorescent species.

## Conclusion

This study presents fluorogenic dyes expanding the spectral properties of FAST to the red edge of the visible spectrum. Originally, FAST emitted green-yellow light upon blue light excitation when using HMBR as a fluorogenic partner. Here, we show that FAST can fluoresce red light upon green light excitation using HBR-3,5DOM instead of HMBR. With FAST, it is thus now possible to adapt the tag color to the spectral conditions of the experiment without changing the protein tag, providing an experimental versatility that is not encountered with autofluorescent proteins. In addition, the ability to change the color by changing the dye renders it possible to switch the color in a single experiment.^20,21^ In this work, we show that the fast exchange dynamics characterizing FAST and the high permeability of the designed fluorogens allow color swapping on the second timescale. We used this unique kinetic signature to develop a method for the selective imaging of intracellular FAST-tagged proteins in spectrally crowded environments. A major obstacle to multiple observations is the spectral overlap between fluorophores, which limits most experiments to the observation of three or four targets. By measuring the degree of temporal anti-correlation of the two fluorescent states of FAST during dynamic color switching, we successfully imaged FAST-tagged proteins in the presence of green and red fluorescent species despite spectral crowding. Imaging three proteins with only two observation channels is a promising step towards overcoming spectral crowding and opens up exciting prospects for pushing the frontiers of multiplexed imaging.

## Materials and methods

### Chemical synthesis—general information

Commercially available reagents were used as the starting materials without further purification. The NMR spectra were recorded on an AC Bruker spectrometer at 300 MHz for ^1^H NMR and 75 MHz for ^13^C NMR; the chemical shifts are reported in ppm with protonated solvent as an internal reference in ^1^H, CHCl_3_ in CDCl_3_ 7.26 ppm, CHD_2_SOCD_3_ in CD_3_SOCD_3_ 2.50 ppm; ^13^C, ^13^CDCl_3_ in CDCl_3_ 77.0 ppm, ^13^CD_3_SOCD_3_ in CD_3_SOCD_3_ 39.5 ppm; the coupling constants *J* are given in Hz. Mass spectroscopy (chemical ionization or high resolution) was performed by the Service de Spectrométrie de Masse de Chimie ParisTech and the Institut de Chimie Organique et Analytique de l’Université d’Orléans. Column chromatography was performed on silica gel 60 (0.040–0.063 nm) Merck. Analytical thin-layer chromatography (TLC) was conducted on Merck silica gel 60 F254 precoated plates.

### Synthesis of 4-hydroxy-3-ethylbenzaldehyde

To a solution of 2-ethylphenol (6.1 g, 50 mmol) in 10% aqueous sodium hydroxide (80 mL, 200 mmol), trichloromethane (15.0 g, 125 mmol) was added dropwise at 60 °C over 1 h, and then the reaction mixture was stirred for 2 h at 60 °C. After cooling, the mixture was neutralized by an aqueous solution of hydrochloric acid and extracted with dichloromethane. The combined organic layers were washed with brine, dried over magnesium sulfate, and concentrated under reduced pressure. The residue was purified by flash chromatography on silica gel with cyclohexane/ethylacetate (7.5/2.5, v/v) to yield the desired 4-hydroxy-3-ethylbenzaldehyde (1.1 g, 15% yield) as a grey pink solid. ^1^H NMR (300 MHz, CDCl_3_, *δ* in ppm): 9.84 (s, 1H), 7.72 (s, 1H), 7.65 (d, *J* = 8.1 Hz, 1H), 6.92 (d, *J* = 8.1 Hz, 1H), 6.51 (s, 1H), 2.70 (q, *J* = 7.5 Hz, 2H), 1.27 (t, *J* = 7.5 Hz, 3H); ^13^C NMR (75 MHz, CDCl_3_, *δ* in ppm): 192.3, 160.5, 131.5, 131.4, 130.6, 129.4, 115.6, 22.8, 13.5; MS (ESI): *m*/*z* 149.2 [M – H]^–^, calcd mass for [C_9_H_9_O_2_]^–^: 149.1; HRMS (ESI): *m*/*z* 149.0608 [M – H]^–^, calcd mass for [C_9_H_9_O_2_]^–^: 149.0603.

### General protocol for the synthesis of HBR-3OM and HBR-2,5DM

A solution containing rhodanine (133 mg, 1.0 mmol) and substituted 4-hydroxy-benzaldehyde (1.0 mmol) in 40 mL of water was stirred at 90 °C for 7 days. After cooling to 4 °C and standing overnight, the precipitate was filtered through a glass filter and the crude solid was washed with water and ethanol and dried over P_2_O_5_, to give the desired product.

#### (*Z*)-5-(4-Hydroxy-3-methoxybenzylidene)-2-thioxo-1,3-thiazolidin-4-one (HBR-3OM)

Yellow powder (52%). ^1^H NMR (300 MHz, CD_3_SOCD_3_, *δ* in ppm): 13.68 (s, 1H), 10.08 (s, 1H), 7.57 (s, 1H), 7.15 (s, 1H), 7.08 (d, *J* = 8.1 Hz, 1H), 6.94 (d, *J* = 8.1 Hz, 1H), 3.83 (s, 3H); ^13^C NMR (75 MHz, CD_3_SOCD_3_, *δ* in ppm): 195.5, 169.5, 150.0, 148.1, 132.8, 125.1, 124.4, 121.2, 116.4, 114.4, 55.7; MS (ESI): *m*/*z* 266.2 [M – H]^–^, calcd mass for [C_11_H_8_NO_3_S_2_]^–^: 266.0; HRMS (ESI): *m*/*z* 265.9951 [M – H]^–^, calcd mass for [C_11_H_8_NO_3_S_2_]^–^: 265.9946.

#### (*Z*)-5-(4-Hydroxy-2,5-dimethylbenzylidene)-2-thioxo-1,3-thiazolidin-4-one (HBR-2,5DM)

Orange powder (38%). ^1^H NMR (300 MHz, CD_3_SOCD_3_, *δ* in ppm): 13.70 (s, 1H), 10.22 (s, 1H), 7.64 (s, 1H), 7.10 (s, 1H), 6.75 (s, 1H), 2.33 (s, 3H), 2.14 (s, 3H); ^13^C NMR (75 MHz, CD_3_SOCD_3_, *δ* in ppm): 196.3, 169.8, 158.9, 140.1, 131.1, 130.0, 123.4, 122.9, 122.0, 117.7, 19.6, 16.1; MS (ESI): *m*/*z* 264.1 [M – H]^–^, calcd mass for [C_12_H_10_NO_2_S_2_]^–^: 264.0; HRMS (ESI): *m*/*z* 264.0158 [M – H]^–^, calcd mass for [C_12_H_10_NO_2_S_2_]^–^: 264.0153.

### General protocol for the synthesis of HBR-3E, HBR-3OE, HBR-3,5DM and HBR-3,5DOM

To a stirred solution of rhodanine (1.5 mmol, 1.0 eq.) and substituted 4-hydroxy-benzaldehyde (1.56 mmol, 1.1 eq.) in 4.5 mL of absolute ethanol, piperidine (1.5 mmol, 1.0 eq.) was added. The solution was stirred at room temperature for 16 h and then neutralized by an aqueous solution of hydrochloric acid. After cooling to 4 °C and standing for 2 h, the precipitate was filtered through a glass filter and the crude solid was washed with water and ethanol and dried over P_2_O_5_, to give the desired product.

#### (*Z*)-5-(3-Ethyl-4-hydroxybenzylidene)-2-thioxothiazolidin-4-one (HBR-3E)

Yellow powder (70%). ^1^H NMR (300 MHz, CD_3_SOCD_3_, *δ* in ppm): 13.67 (s, 1H), 10.35 (s, 1H), 7.54 (s, 1H), 7.30 (m, 2H), 6.94 (d, *J* = 8.1 Hz, 1H), 2.57 (q, *J* = 7.5 Hz, 2H), 1.15 (t, *J* = 7.5 Hz, 3H); ^13^C NMR (75 MHz, CD_3_SOCD_3_, *δ* in ppm): 195.5, 169.4, 158.3, 132.8, 132.0, 131.3, 130.6, 124.0, 120.5, 115.8, 22.5, 13.7; MS (ESI): *m*/*z* 264.3 [M – H]^–^, calcd mass for [C_12_H_10_NO_2_S_2_]^–^: 264.0; HRMS (ESI): *m*/*z* 264.0157 [M – H]^–^, calcd mass for [C_12_H_10_NO_2_S_2_]^–^: 264.0153.

#### (*Z*)-5-(3-Ethoxy-4-hydroxybenzylidene)-2-thioxothiazolidin-4-one (HBR-3OE)

Orange powder (74%). ^1^H NMR (300 MHz, CD_3_SOCD_3_, *δ* in ppm): 13.69 (s, 1H), 10.00 (s, 1H), 7.55 (s, 1H), 7.12 (d, *J* = 1.8 Hz, 1H), 7.07 (dd, *J* = 1.8 Hz, 8.4 Hz, 1H), 6.95 (d, *J* = 8.1 Hz, 1H), 4.08 (q, *J* = 6.9 Hz, 2H), 1.36 (t, *J* = 6.9 Hz, 3H); ^13^C NMR (75 MHz, CD_3_SOCD_3_, *δ* in ppm): 195.4, 169.4, 150.2, 147.3, 132.8, 125.1, 124.4, 121.0, 116.4, 115.4, 63.9, 14.6; MS (ESI): *m*/*z* 280.1 [M – H]^–^, calcd mass for [C_12_H_10_NO_3_S_2_]^–^: 280.0; HRMS (ESI): *m*/*z* 280.0108 [M – H]^–^, calcd mass for [C_12_H_10_NO_3_S_2_]^–^: 280.0102.

#### (*Z*)-5-(4-Hydroxy-3,5-dimethylbenzylidene)-2-thioxothiazolidin-4-one (HBR-3,5DM)

Yellow powder (73%). ^1^H NMR (300 MHz, CD_3_SOCD_3_, *δ* in ppm): 13.67 (s, 1H), 9.26 (s, 1H), 7.45 (s, 1H), 7.17 (s, 2H), 2.21 (s, 6H); ^13^C NMR (75 MHz, CD_3_SOCD_3_, *δ* in ppm): 195.6, 169.5, 156.6, 132.6, 131.5 (2C), 125.4 (2C), 124.0, 120.8, 16.6 (2C); MS (ESI): *m*/*z* 264.3 [M – H]^–^, calcd mass for [C_12_H_10_NO_2_S_2_]^–^: 264.0; HRMS (ESI): *m*/*z* 264.0158 [M – H]^–^, calcd mass for [C_12_H_10_NO_2_S_2_]^–^: 264.0153.

#### (*Z*)-5-(4-Hydroxy-3,5-dimethoxybenzylidene)-2-thioxothiazolidin-4-one (HBR-3,5DOM)

Brown powder (72%). ^1^H NMR (300 MHz, CD_3_SOCD_3_, *δ* in ppm): 13.71 (s, 1H), 9.46 (s, 1H), 7.57 (s, 1H), 6.88 (s, 2H), 3.83 (s, 6H); ^13^C NMR (75 MHz, CD_3_SOCD_3_, *δ* in ppm): 195.3, 169.3, 148.3 (2C), 139.2, 133.0, 123.2, 121.4, 108.5 (2C), 56.1 (2C); MS (ESI): *m*/*z* 296.1 [M – H]^–^, calcd mass for [C_12_H_10_NO_4_S_2_]^–^: 296.0; HRMS (ESI): *m*/*z* 296.0056 [M – H]^–^, calcd mass for [C_12_H_10_NO_4_S_2_]^–^: 296.0051.

### Physical chemistry experiments

pH measurements were performed on a standard pH meter PHM210 Radiometer Analytical (calibrated with aqueous buffers at pH 4 and 7 or 10) with a Crison 5208 Electrode (Barcelona, Spain). The UV/Vis absorption spectra were recorded with 1 cm × 1 cm quartz cuvettes (Hellma) on a diode array UV/Vis spectrophotometer (Evolution array, Thermo Scientific). The corrected fluorescence spectra upon one-photon excitation were recorded with a Photon Technology International QuantaMaster QM-1 spectrofluorimeter (PTI, Monmouth Junction, NJ) equipped with a Peltier cell holder (TLC50, Quantum Northwest, Shoreline, WA). The overall emission quantum yields after one-photon excitation *φ* were determined as previously described.^14^ The affinity constants were determined by spectrofluorometric titration using a Spark 10M plate reader (Tecan) following the protocols previously described.^14^

### Molecular biology

FAST is a variant of the photoactive yellow protein (PYP) containing the mutations C69G, Y94W, T95M, F96I, D97P, Y98T, Q99S, M100R, and T101G. The construction of plasmid pAG87 for the bacterial expression of FAST fused to a His-tag was previously described.^14^ The construction of the plasmids pAG104, pAG109 and pAG106 for the mammalian expression of FAST, FAST fused to zebrafish H2B (H2B-FAST), and FAST fused to the mitochondrial targeting sequence (MTS) from subunit VIII of human cytochrome c oxidase (MTS-FAST) was previously described.^14^ Plasmid pAG28 for the mammalian expression of EGFP fused to lyn N-terminal domain (lyn) for inner membrane anchoring was previously described.^18^ The plasmids CV2307 and CV2326 for the mammalian expression of mCherry fused to the membrane anchoring (lyn) peptide from the mouse tyrosine-protein kinase lyn and to the N-terminal 25 amino acid mitochondrial targeting signal (MTS) of COX4 from *S. cerevisiae* were assembled using MXS chaining by sequentially inserting a CMV promoter, and the sequences of the lyn (MGCIKSKRKDVEN) or MTS (MLSLRQSIRFFKPATRTLCSSRYLL) peptide tags in a plasmid containing the mCherry coding sequence coupled to the bovine growth hormone poly A signal.

### Bacterial expression and protein purification

pAG87 was transformed in Rosetta(DE3)pLysS *E. coli* (New England Biolabs). The cells were grown at 37 °C in Lysogeny Broth (LB) medium complemented with 50 μg mL^–1^ kanamycin and 34 μg mL^–1^ chloramphenicol to OD_600 nm_ 0.6. Expression was induced for 4 h by adding isopropyl β-d-1-thiogalactopyranoside (IPTG) to a final concentration of 1 mM. The cells were harvested by centrifugation (6000 × *g* for 15 min at 4 °C) and frozen. The cell pellet was resuspended in lysis buffer (phosphate buffer 50 mM, NaCl 150 mM, MgCl_2_ 2.5 mM, protease inhibitor, DNase, pH 7.4) and sonicated (5 min at 20% of amplitude). The lysate was incubated for 2 h at 4 °C to allow DNA digestion by DNase. The cellular fragments were removed by centrifugation (15 000 × *g* for 1 h at 4 °C). The supernatant was incubated overnight at 4 °C under gentle agitation with Ni-NTA agarose beads in phosphate buffered saline (PBS) (sodium phosphate 50 mM, NaCl 150 mM, pH 7.4) complemented with 10 mM imidazole. The beads were washed with 20 volumes of PBS containing 20 mM imidazole, and with 5 volumes of PBS complemented with 40 mM imidazole. His-tagged proteins were eluted with 5 volumes of PBS complemented with 0.5 M imidazole, followed by dialysis with PBS.

### Mammalian cell culture

The HeLa cells were cultured in DMEM supplemented with phenol red, Glutamax I, 10% (v/v) fetal calf serum (FCS), and 1% penicillin–streptomycin at 37 °C within a 5% CO_2_ atmosphere. For microscopic imaging, the cells were seeded in μDish or μSlide IBIDI (Biovalley) coated with poly-l-lysine. The cells were transiently transfected using Genejuice (Merck) according to the manufacturer’s protocol. The cells were washed with PBS, and treated with DMEM media (without serum and phenol red) containing the fluorogens at the indicated concentration. The cells were imaged directly without washing.

### Cell viability assay

The HeLa cells were treated with DMEM media containing the fluorogens at the indicated concentrations for the indicated times. The cell viability was evaluated by fluorescence microscopy using the LIVE/DEAD® viability/cytotoxicity assay kit (Molecular Probes, Life Technologies) following the manufacturer’s protocol.

### Fluorescence microscopy

The confocal micrographs were acquired on a Zeiss LSM 710 Laser Scanning Microscope equipped with a Plan Apochromat 63×/1.4 NA oil immersion objective. ZEN software was used to collect the data. The images were analyzed with Image J.

### Two-color cross-correlation analysis

The two-color cross-correlated images were obtained as follows. A movie was acquired in the green and red (g: green and r: red) fluorescence channels over the evolution duration observed after adding HMBR fluorogen to the cellular medium. The cross-correlated image was then computed from the movie as 
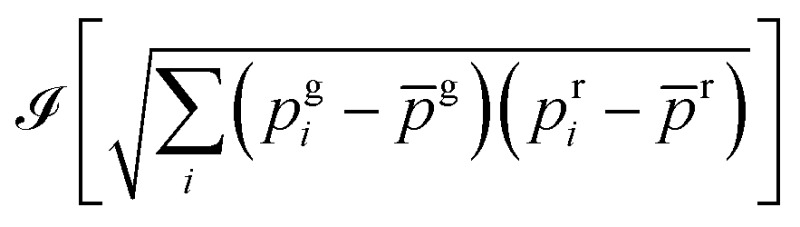
 where ℐ takes the imaginary part of the FAST concentration proportional square root function, *i* denotes the frame number, *p*g*i* (*p*r*i*) is the fluorescence intensity of pixel *p* in the green (red) channel of the image *i*, and *p* ^g^ (*p* ^r^) denotes the average fluorescence intensity of pixel *p* over the whole movie duration in the green (red) channel. Eventually a median filter (3 × 3 pixel^2^) was applied to yield the final image.
