# Supplementary material for: Dynamic multicolor protein labeling in living cells
Source: Chem Sci. 2017 May 30;8(8):5598–605. doi: 10.1039/c7sc01364g (PMC5618792; doi:10.1039/c7sc01364g)

Electronic Supplementary Material (ESI) for Chemical Science.
This journal is © The Royal Society of Chemistry 2017

SUPPORTING INFORMATION

Dynamic multi-color protein labeling in living cells

Chenge Li[a,b], Marie-Aude Plamont[a,b], Hanna L. Sladitschek[c], Vanessa Rodrigues[a,b], Isabelle Aujard[a,b], Pierre Neveu[c], Thomas Le Saux[a,b], Ludovic Jullien[a,b,*] & Arnaud Gautier[a,b,*]

[a] École Normale Supérieure, PSL Research University, UPMC Univ Paris 06, CNRS, Département de Chimie, PASTEUR, 24 rue Lhomond, 75005 Paris, France

[b] Sorbonne Universités, UPMC Univ Paris 06, ENS, CNRS, PASTEUR, 75005 Paris, France

[c] Cell Biology and Biophysics Unit, European Molecular Biology Laboratory, Meyerhofstr. 1, D-69117 Heidelberg

* Correspondence should be addressed to: ludovic.jullien@ens.fr and arnaud.gautier@ens.fr

LEGENDS OF SUPPLEMENTARY MOVIES

Movie S1. Dynamic color switching. Confocal time-lapse of a live HeLa cell expressing H2B-FAST labeled with 5 µM of HBR-3,5DOM upon replacement of the medium with solution containing 5 µM of HMBR (Single excitation at 488 nm; HMBR emission channel 493-538 nm; HBR-3,5-DOM emission channel 649-797 nm). HMBR was added at t = 0 s. Cells were grown in a minifluidic channel enabling easy solution replacement. See also **Figure 3C**.

Movie S2. Dynamic color switching in a spectrally crowded environment. Confocal time-lapse of live HeLa cells co-expressing lyn-EGFP (membrane), MTS-mCherry (mitochondria) and H2B-FAST (nucleus) upon fluorogen exchange (Green channel Ex/Em 488/493-575 nm; Red channel Ex/Em 543/578-797 nm). Cells were initially stained with 5 µM HBR-3,5DOM. Fluorogen exchange was induced by addition of an excess of HMBR at t = 0 s. The final concentrations of HBR-3,5DOM and HMBR were respectively 0.83 µM and 4.2 µM. See also **Figure S4**.

SUPPLEMENTARY FIGURES

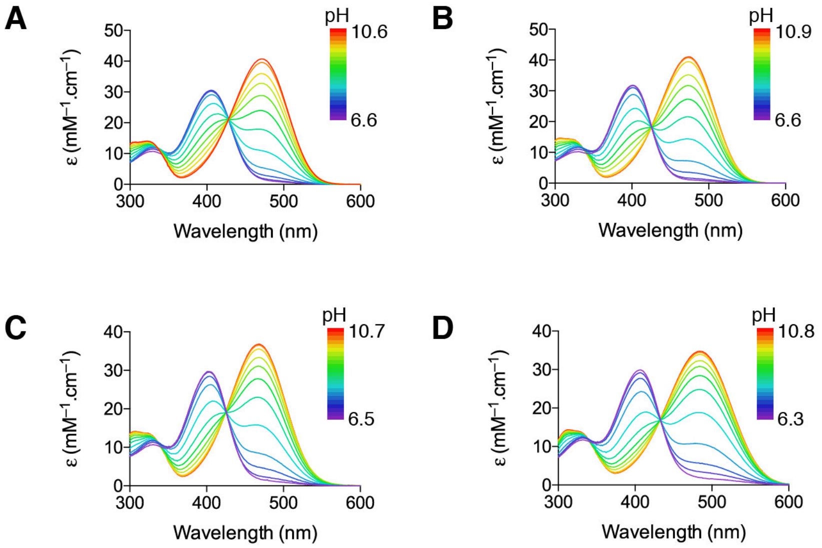

Figure S1. Absorption spectra of HBR-2,5DM (A), HBR-3,5DM (B), HBR-3OM (C) and HBR-3,5DOM (D) in solution in function of pH. The spectra were recorded in 0.04 M Britton–Robinson buffer (0.1 M ionic strength) at 25°C.

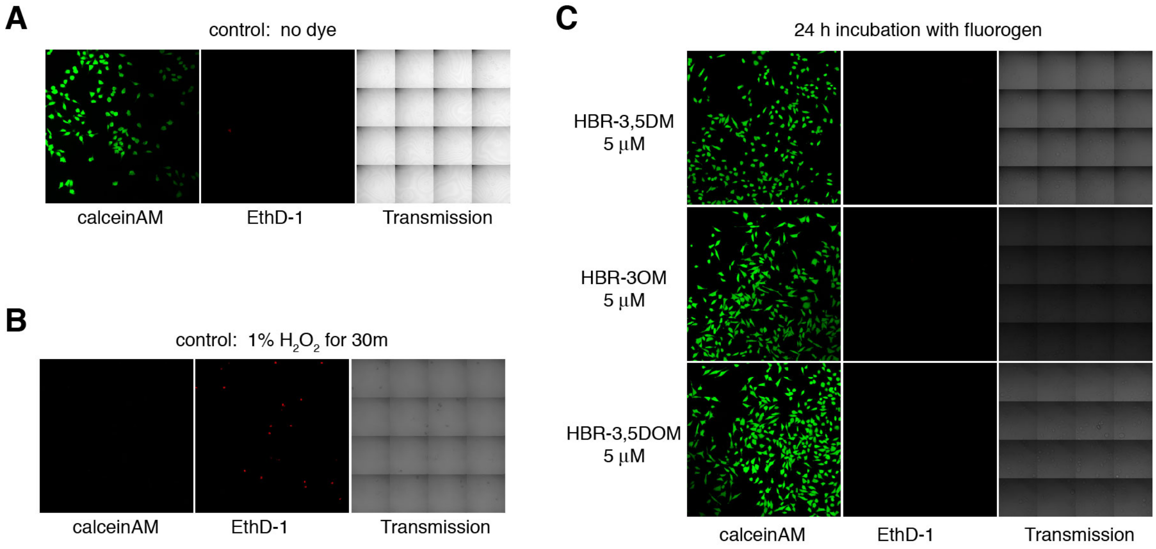

Figure S2. Viability assay of HeLa cells incubated for 24 h with solutions of HBR-3,5DM, HBR-3OM and HBR-3,5DOM at 5 µM. Cell viability was tested by using calceinAM and EthD1 (LIVE/DEAD® viability/cytotoxicity assay kit). CalceinAM is a cell-permeant profluorophore cleaved by intracellular esterases releasing the green fluorescent polyanionic calcein in live cells. EthD1 (Ethidium homodimer 1) is a non cell-permeant nucleic acid red fluorescent stain that enters only cells with damaged membranes and undergoes a fluorescence enhancement upon binding to nucleic acids, thereby producing a bright red fluorescence in dead cells. Control experiments with HeLa cells non-incubated with dye (**A**) or incubated for 30 min with 1% hydrogen peroxyde (**B**) are shown. Cell fluorescence was evaluated by confocal microscopy. The experiment in (**C**) shows that HBR-3,5DM, HBR-3OM and HBR-3,5DOM are non-toxic for HeLa cells at the concentrations used for imaging.

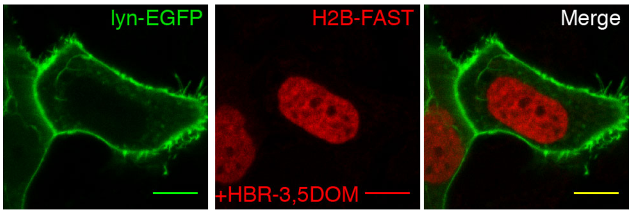

Figure S3. Dual-color imaging with a single excitation. Confocal micrographs of live HeLa cells co-expressing lyn-EGFP and H2B-FAST labeled with 5 μM HBR-3,5DOM upon single excitation at 488 nm (Green emission channel 493-538 nm; Red emission channel 600-797 nm). Scale bars 10 μm.

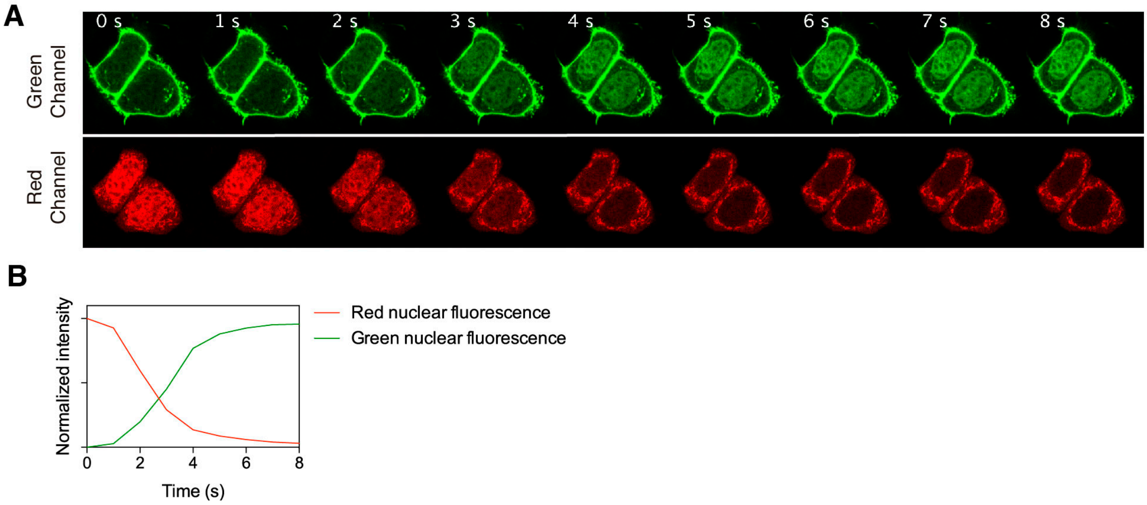

Figure S4. Dynamic color switching in spectrally crowded environment. (A) Time series of live HeLa cells co-expressing lyn-EGFP (membrane), MTS-mCherry (mitochondria) and H2B-FAST (nucleus) upon fluorogen exchange (Green channel Ex/Em 488/493-575 nm; Red channel Ex/Em 543/578-797 nm). Cells were initially stained with 5 µM HBR-3,5DOM. Fluorogen exchange was induced by addition of an excess of HMBR at t = 0 s. The final concentrations of HBR-3,5DOM and HMBR were respectively 0.83 µM and 4.2 µM. See also **Movie S2**. **(B)** Temporal evolution of the green and red nuclear fluorescence intensities.

SUPPLEMENTARY TABLES

Table S1. Physico-chemical properties of HMBR, HBR-3,5DM, HBR-2,5DM, HBR-3OM, HBR-3,5DOM in aqueous solutions. Abbreviations are as follows : pK_A, acidity constant ; $\lambda_{abs,neutral}$, wavelength of maximal absorption of the protonated state; $\lambda_{abs,anionic}$, wavelength of maximal absorption of the anionic state.

Fluorogen	pK_A	$\lambda_{abs,neutral}$ (nm)	$\lambda_{abs,anionic}$ (nm)
HMBR	8.7	401	461
HBR-3,5DM	8.7	401	473
HBR-2,5DM	8.7	406	472
HBR-3OM	8.3	403	468
HBR-3,5DOM	8.3	407	484

(Z)-5-(4-hydroxy-3-methoxybenzylidene)-2-thioxo-1,3-thiazolidin-4-one (HBR-3OM)

^{1}H

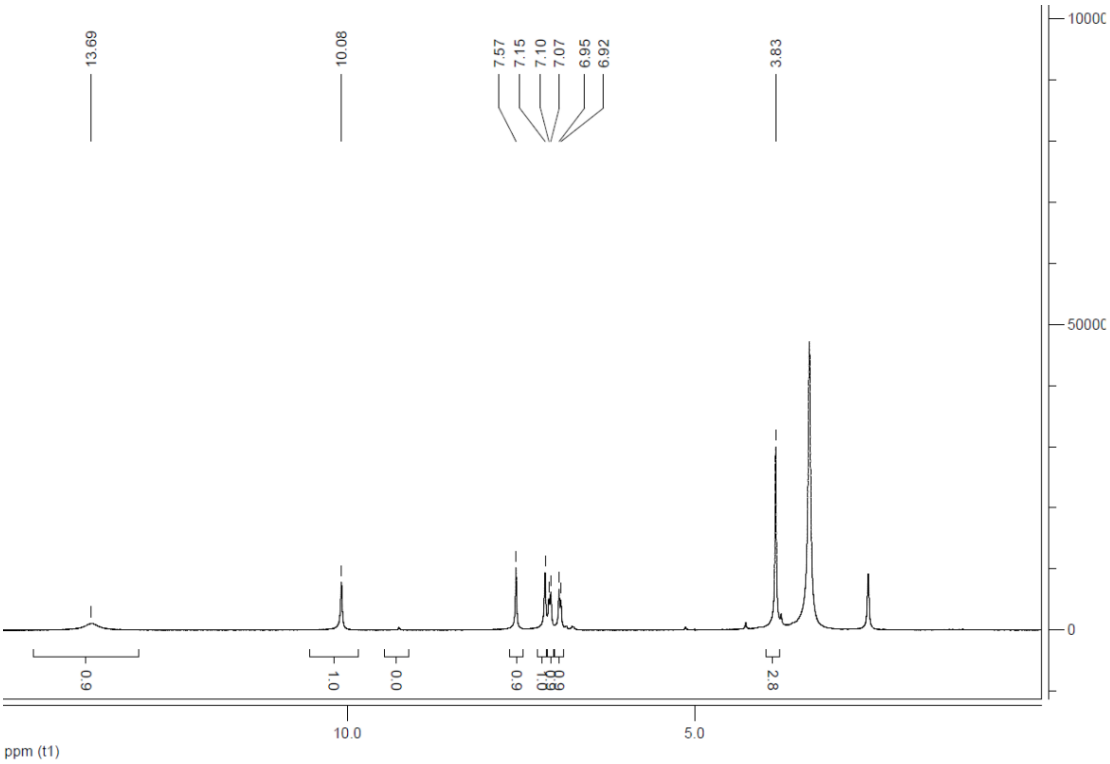

^{13}C

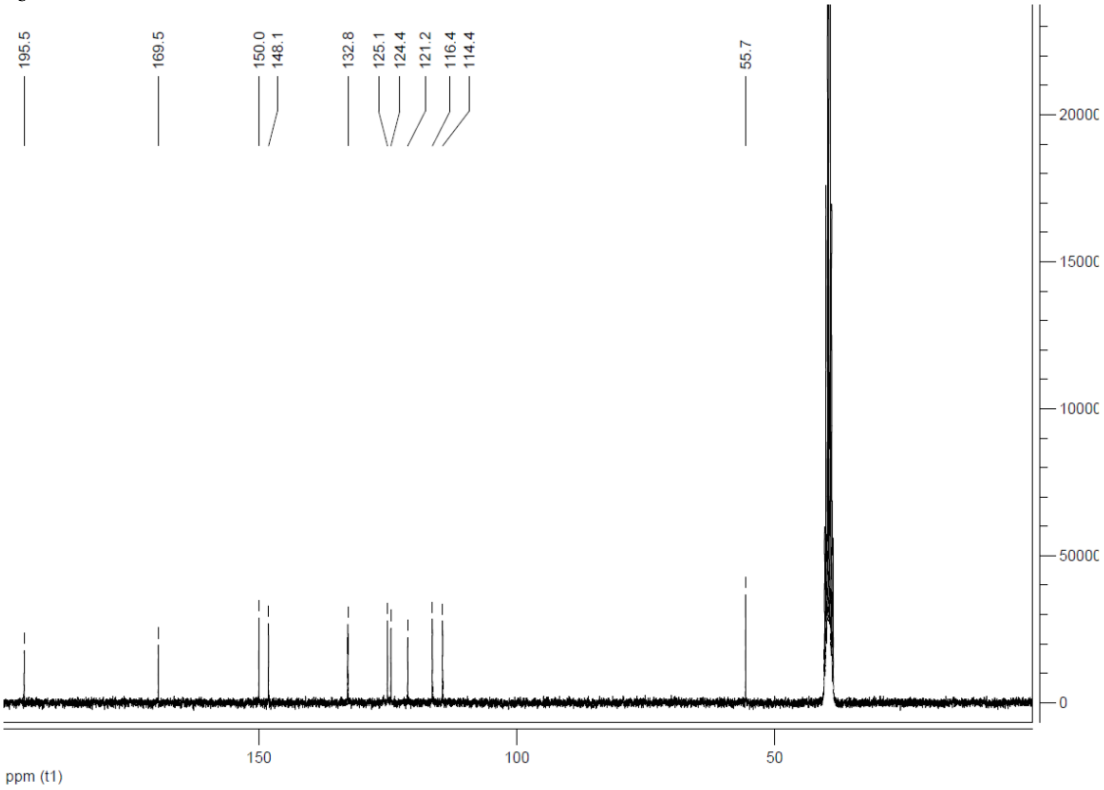

(Z)-5-(4-hydroxy-2, 5-dimethylbenzylidene)-2-thioxo-1,3-thiazolidin-4-one (HBR-2,5DM)

^{1}H

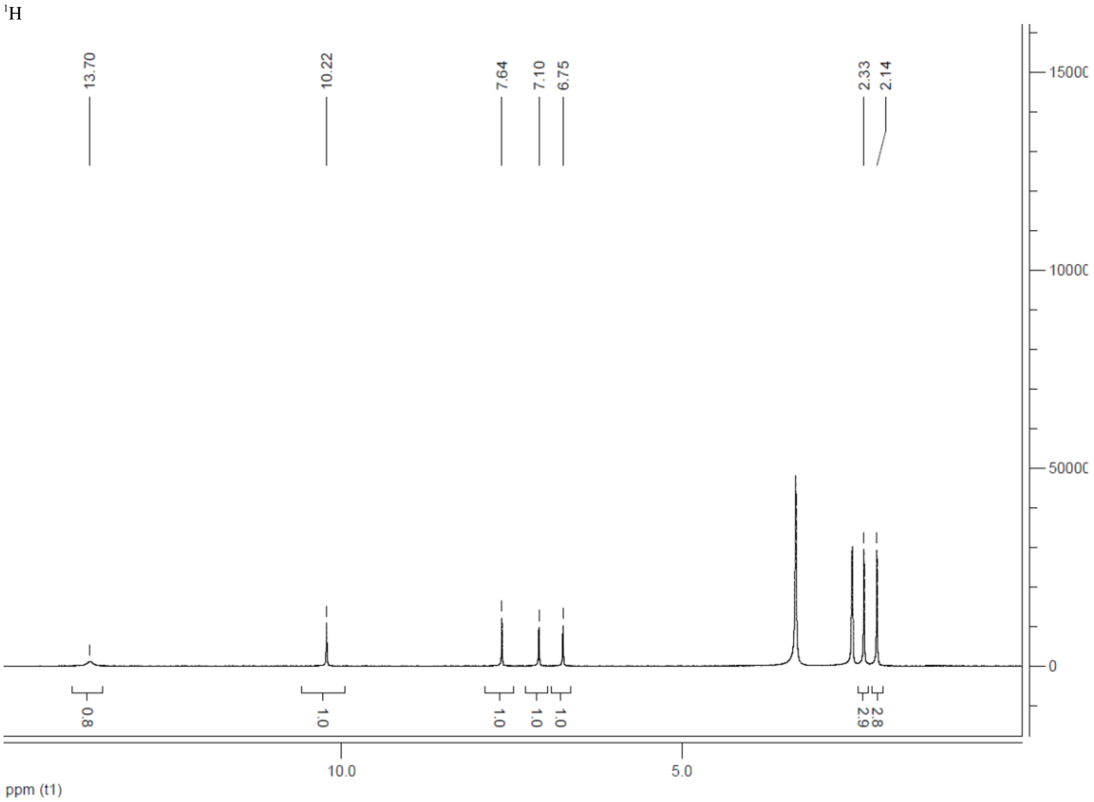

^{13}C

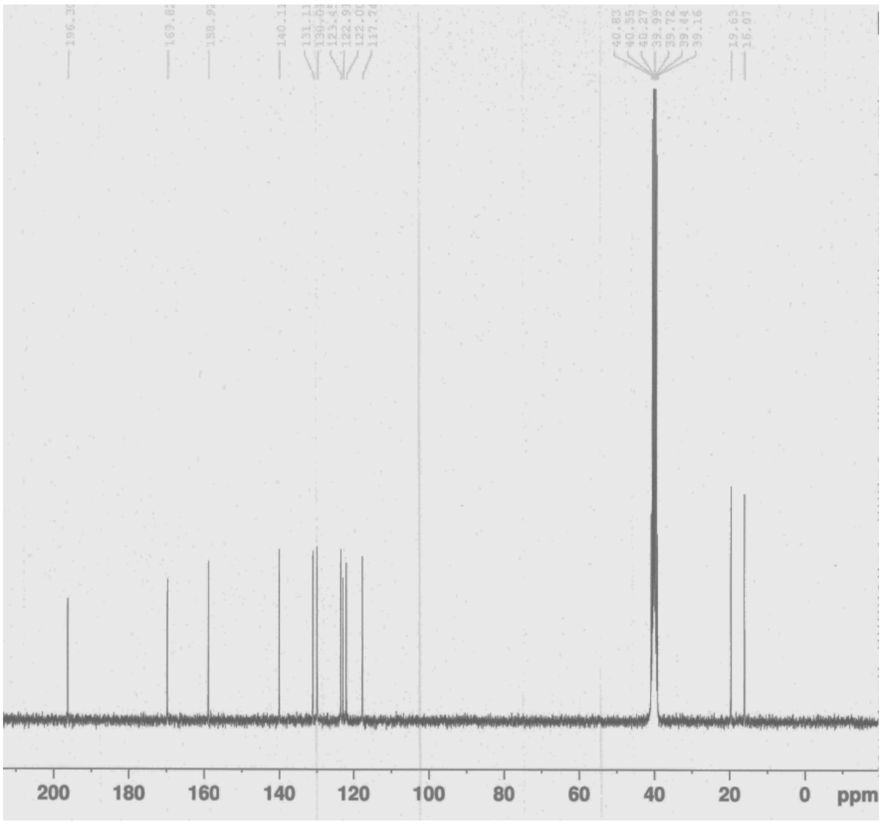

(Z)-5-(3-ethyl-4-hydroxybenzylidene)-2-thioxothiazolidin-4-one (HBR-3E)

^{1}H

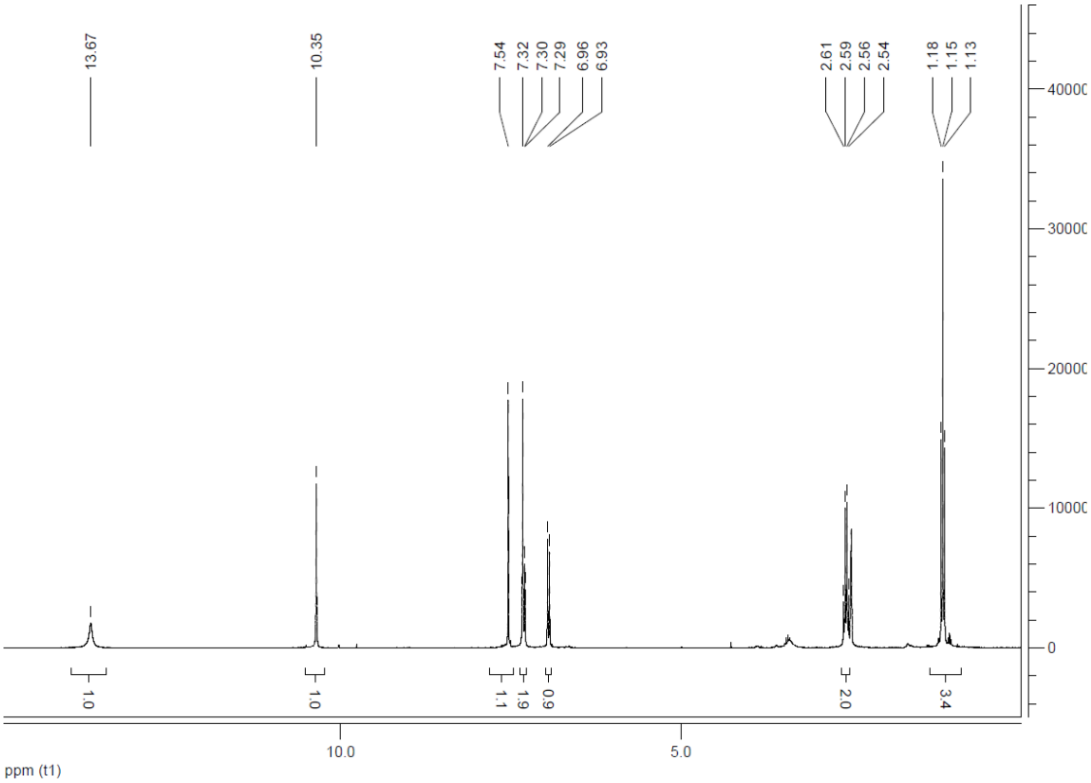

^{13}C

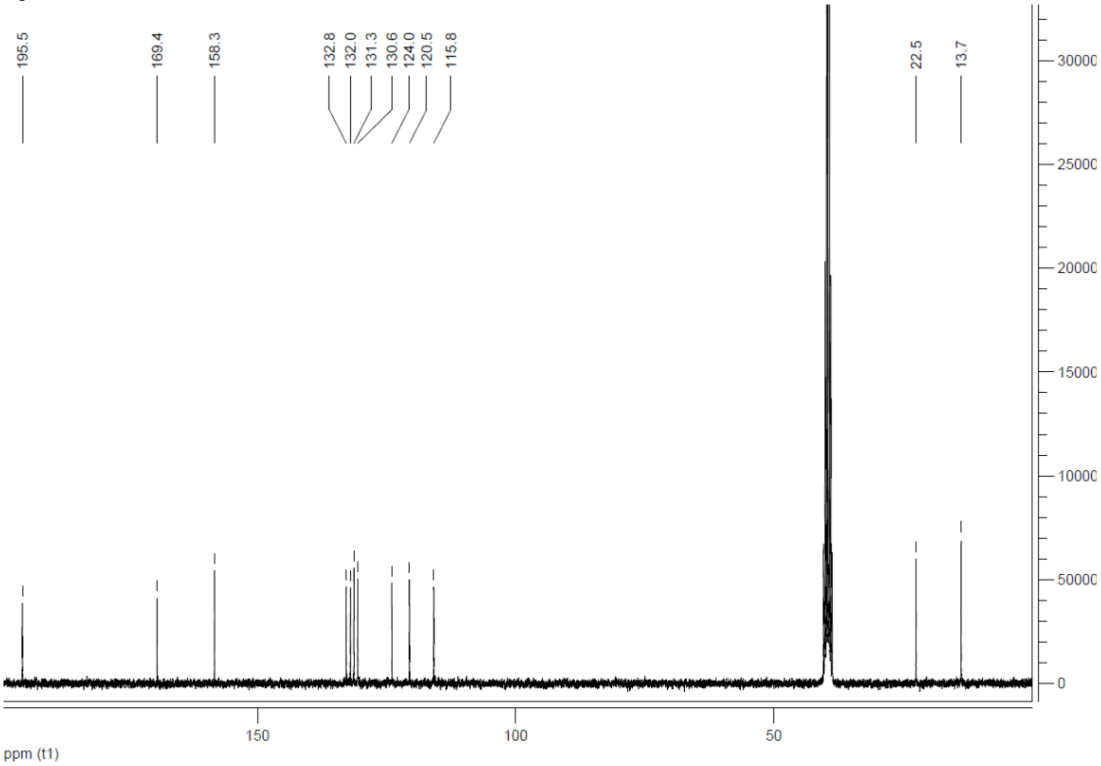

(Z)-5-(3-ethoxy-4-hydroxybenzylidene)-2-thioxothiazolidin-4-one (HBR-3OE)

^{1}H

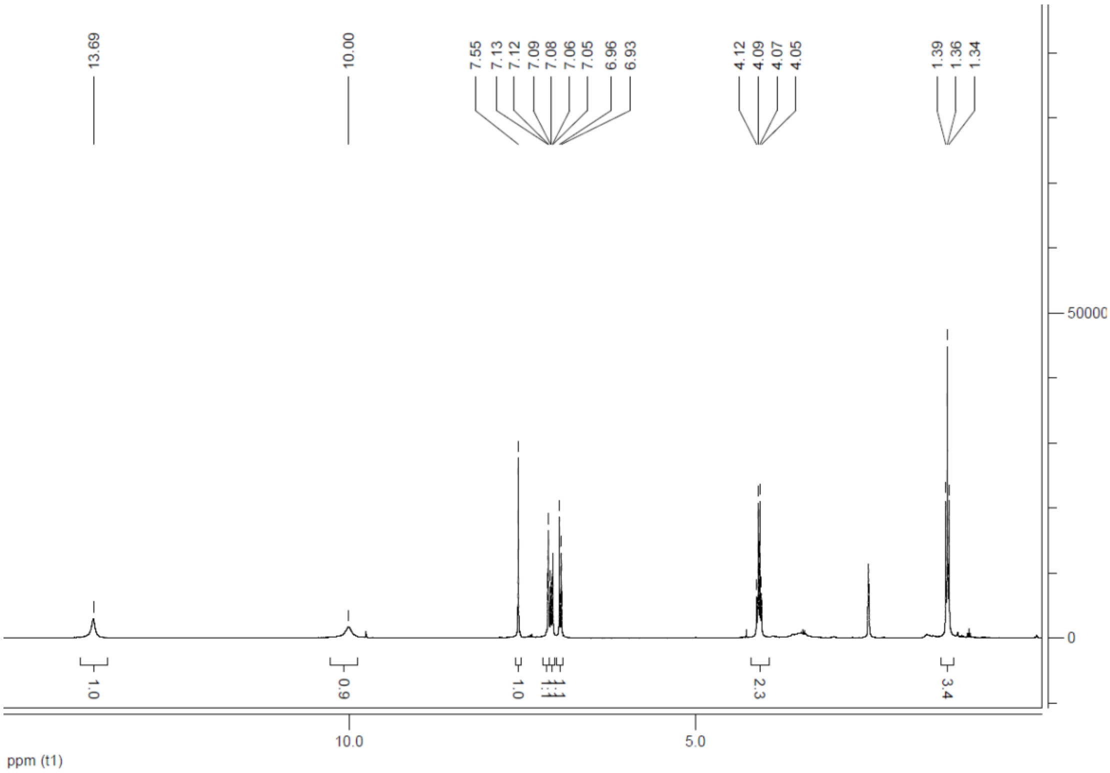

^{13}C

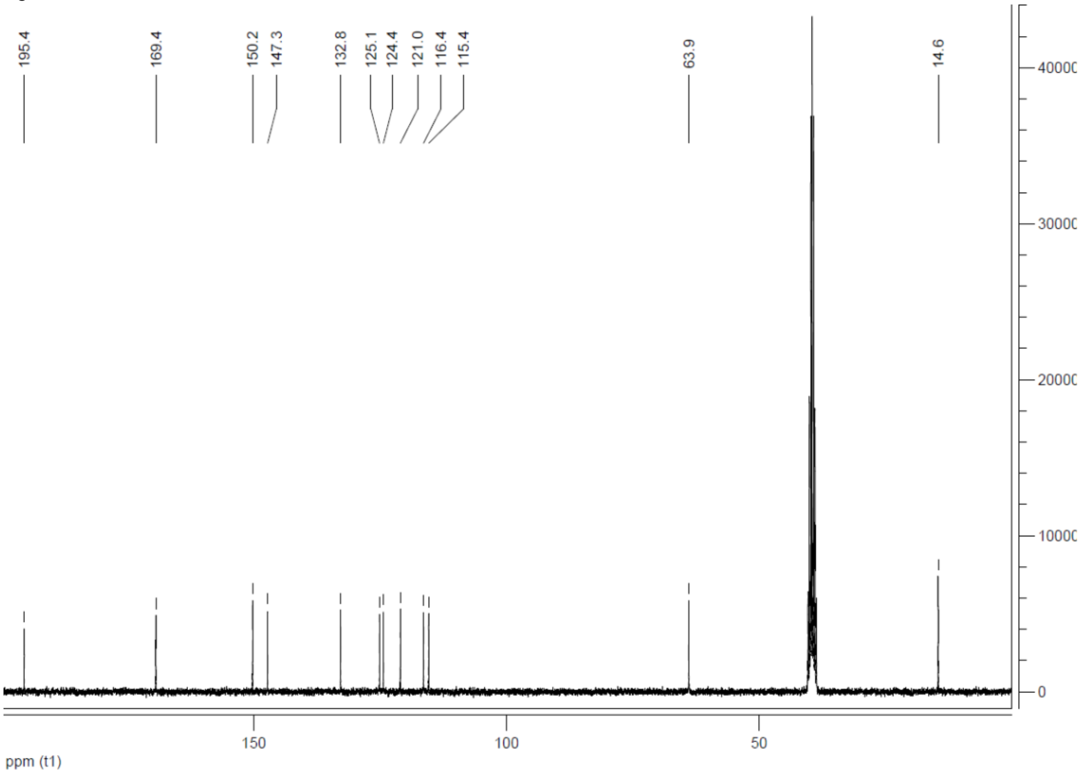

(Z)-5-(4-hydroxy-3,5-dimethylbenzylidene)-2-thioxothiazolidin-4-one (HBR-3,5DM)

^{1}H

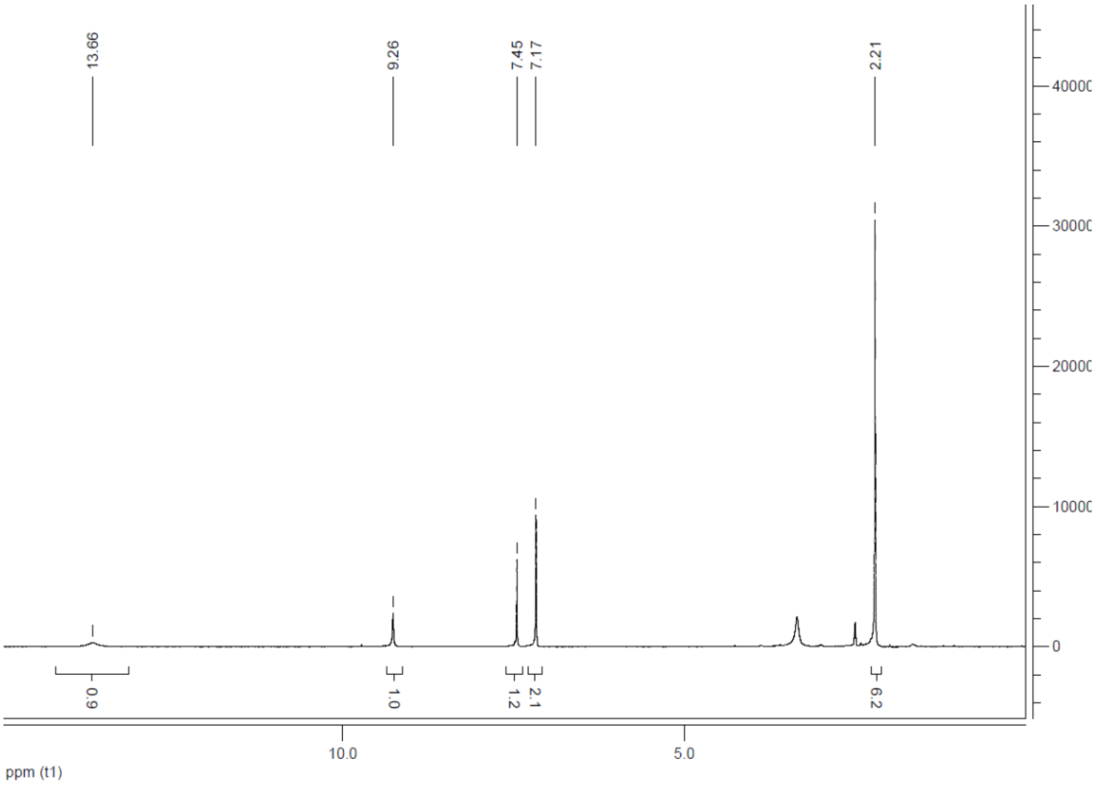

^{13}C

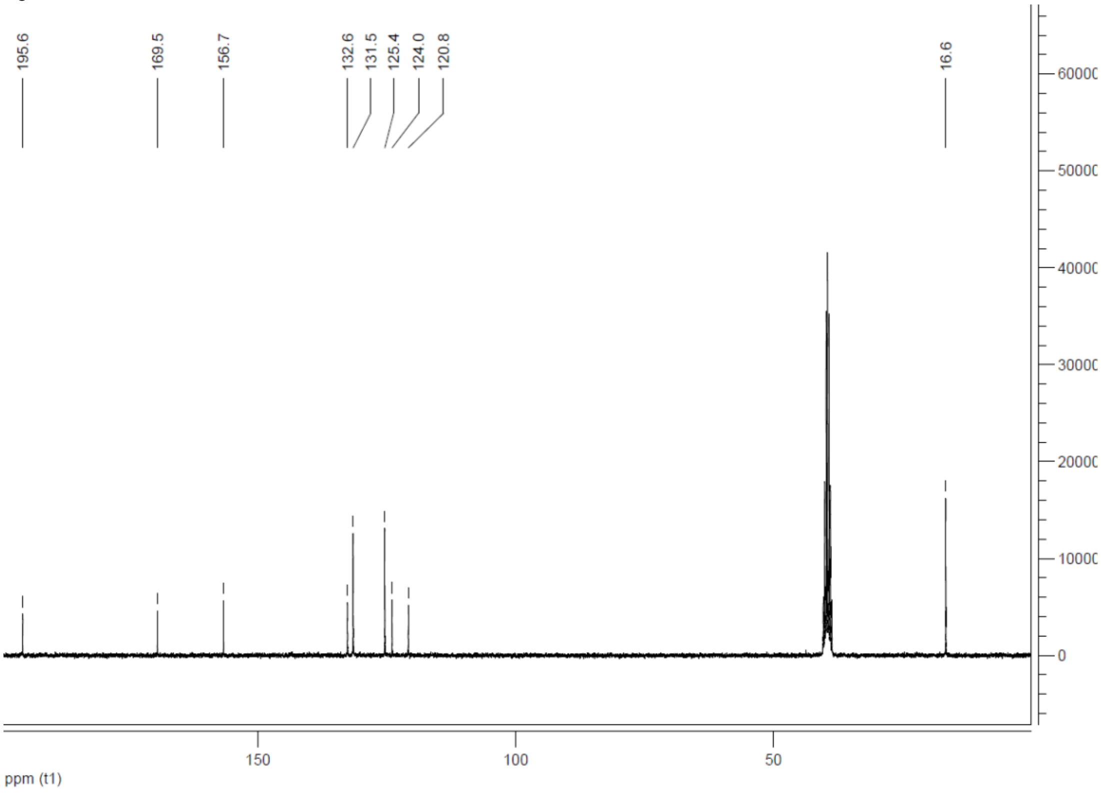

(Z)-5-(4-hydroxy-3,5-dimethoxybenzylidene)-2-thioxothiazolidin-4-one (HBR-3,5DOM)

^{1}H

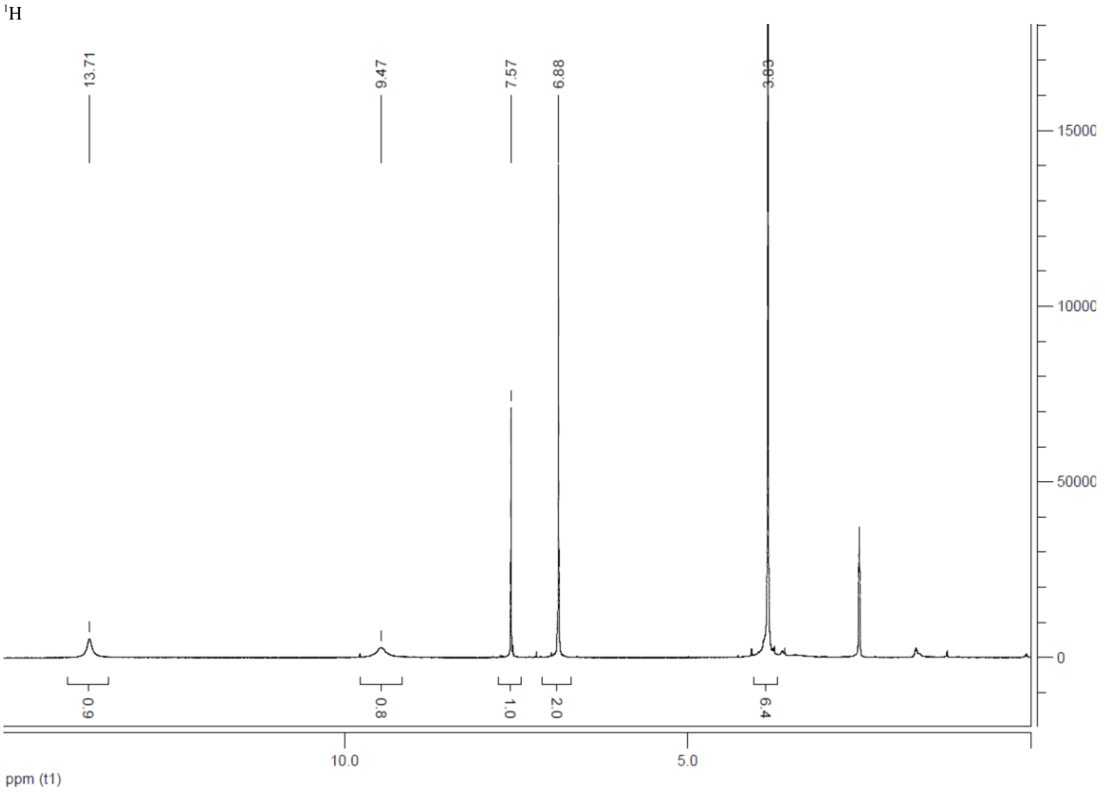

^{13}C

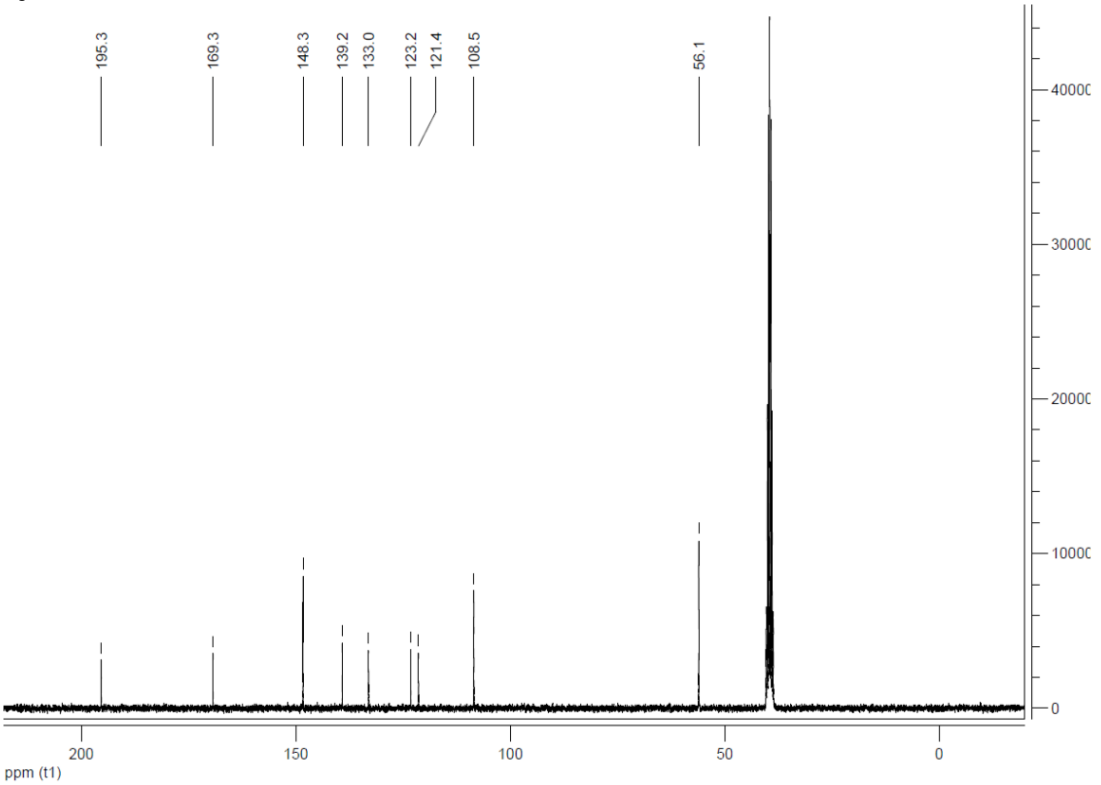

Supplement: Supplementary file 3 [file SC-008-C7SC01364G-s003.pdf]
